# A novel nomogram for predicting cancer-specific survival in women with uterine sarcoma: a large population-based study

**DOI:** 10.1186/s12905-022-01739-5

**Published:** 2022-05-14

**Authors:** Yuan-jie Li, Jun Lyu, Chen Li, Hai-rong He, Jin-feng Wang, Yue-ling Wang, Jing Fang, Jing Ji

**Affiliations:** 1 Department of Human Anatomy, Histology and Embryology, School of Basic Medical Sciences, Xi’an Jiao Tong University Health Science Center, Xi’an, Shaanxi China; 2 Department of Clinical Research, The First Affiliated Hospital of Jinan University, Guangzhou, Guangdong China; 3 Department of Gynecology and Obstetrics, The First Affiliated Hospital of Xi’an Jiaotong University, Xi’an, 710061 Shaanxi China; 4 Department of Clinical Research Center, The First Affiliated Hospital of Xi’an Jiaotong University, Xi’an, Shaanxi China

**Keywords:** Nomogram, Uterine sarcoma, SEER database, Cancer-specific survival

## Abstract

**Background:**

Uterine sarcoma (US) is a rare malignant uterine tumor with aggressive behavior and rapid progression. The purpose of this study was to constructa comprehensive nomogram to predict cancer-specific survival (CSS) of patients with US-based on the Surveillance, Epidemiology, and End Results (SEER) database.

**Methods:**

A retrospective population-based study was conducted using data from patients with US between 2010 and 2015 from the SEER database. They were randomly divided into a training cohort and a validation cohort ata 7-to-3 ratio. Multivariate Cox analysis was performed to identify independent prognostic factors. Subsequently, a nomogram was established to predict patient CSS. The discrimination and calibration of the nomogram were evaluated by the concordance index (C-index) and the area under the curve (AUC). Finally, net reclassification improvement (NRI), integrated discrimination improvement (IDI), calibration plotting, and decision-curve analysis (DCA) were used to evaluate the benefits of the new prediction model.

**Results:**

A total of 3861 patients with US were included in our study. As revealed in multivariate Cox analysis, age at diagnosis, race, marital status, insurance record, tumor size, pathology grade, histological type, SEER stage, AJCC stage, surgery status, radiotherapy status, and chemotherapy status were found to be independent prognostic factors. In our nomogram, pathology grade had strongest correlation with CSS, followed by age at diagnosis and surgery status. Compared to the AJCC staging system, the new nomogram showed better predictive discrimination with a higher C-index in the training and validation cohorts (0.796 and 0.767 vs. 0.706 and 0.713, respectively). Furthermore, the AUC value, calibration plotting, NRI, IDI, and DCA also demonstrated better performance than the traditional system.

**Conclusion:**

Our study validated the first comprehensive nomogram for US, which could provide more accurate and individualized survival predictions for US patients in clinical practice.

## Background

Uterine sarcoma (US) is a rare malignant uterine tumor that accounts for 3–7% of all uterine cancer cases [1] and is characterized by aggressive behavior and rapid progression. The incidence of US ranges from 1.55 to 1.95 per 100,000 women per year [2]. The current classification of US includes endometrial stromal sarcoma, leiomyosarcoma, and mixed epithelial and mesenchymal tumors according to the common histological types [3]. No common etiology has been identified, but several agents might be associated with US, such as tamoxifen treatment, pelvic radiation therapy, and hereditary leiomyomatosis [4]. Management involves coordinating multidisciplinary treatment including surgery, radiotherapy, chemotherapy and hormonal blockade. However, the 5-year survival rate is less than 50% in early stages and less than 15% in advanced stages [5, 6].

The American Joint Committee on Cancer (AJCC) staging system is the most extensively used clinical tool in determining the prognosis of cancer [7] and it is based on the tumor (T), number of metastatic lymph nodes (N), and distant metastasis (M). However, US is a very heterogeneous disease. The patient response to therapy differs widely, and the survival rate varies within the same stage. Some clinical characteristics, such as age, race, and tumor size, are noteworthy factors influencing the individual survival outcomes of cancer patients [8, 9]. For example, US is twice asfrequent among black women than among white women, and the risk of sarcoma is higher among women aged over 50 years [10]. Thus, a novel, more accurate prognostic tool that includes personalized characteristics is necessary to improve the accuracy of prognosis among women with US.

Nomograms presented by graphs have become widely used to predict the outcome of malignant tumors. This study aimed to develop an effective nomogram to predict the cancer-specific survival (CSS) of US patients based on a cohort from the SEER database to help guide individual treatment decisions for US patients.

## Methods

### Study design and data source

This is a retrospective population-based study of the data from patients diagnosed with US between 2010 and 2015, obtained from the SEER database. The SEER data are registered cases of cancer from throughout the USA.

All of the related information was derived from the SEER program (the latest version covering 18 registries with additional chemotherapy data) by using SEER*Stat version 8.3.6.1 (https://seer.cancer.gov/) [11–13]. The population included in this study was patients diagnosed with uterine sarcoma between 2010 and 2015, with the ICD-O-3 morphology codes [10] 8800/3–8805/3; 8890/3, 8891/3, 8896/3; 8933/3; 8930/3, 8931/3, 8935/3; 8950/3, 8951/3, and 8980/3. We initially excluded other morphological US and selected 3,922 female patients between 2010 and 2015.

### Data collection

Demographic and clinicopathological features were extracted from SEER, including race, age at diagnosis, marital status, tumor size, pathology grade, SEER stage, AJCC stage, surgery, chemotherapy, and radiotherapy. We noticed that the summary stage in the SEER database had four levels: in situ, localized, regional, and distant. In our study, we only included the last three levels due to the lack of patient data for the in situ category. Tumor pathology grade wasdivided into four levels according to the degree of differentiation: Grade I (well), II (moderately), III (poorly), and IV (undifferentiated or anaplastic) [12]. The tumor size was classified as ≤ 50 mm, > 50 mm and unknown. Based on the SEER database, surgery status was divided into two groups: “Yes” means surgery performed, while “No” means no surgery performed due to three situations as follows: not recommended, recommended but the patient refused or died before surgery. Radiotherapy status was also classified: “Yes” means any type of radiation performed, including radioactive implants, beam radiation, or a combination of beams with implants or isotopes [12], while “No” means none/unknown, refused, or recommended but unknown if administered. Chemotherapy status was classified as follows: “Yes” means chemotherapy performed, and “No” means not performed or unknown. The endpoint of this study was death of the patients due to US. We excluded one patient who was found to have no tumor, as well as those who had an unknown insurance record (n = 60).

### Criteria for data selection

**Fig. 1:**
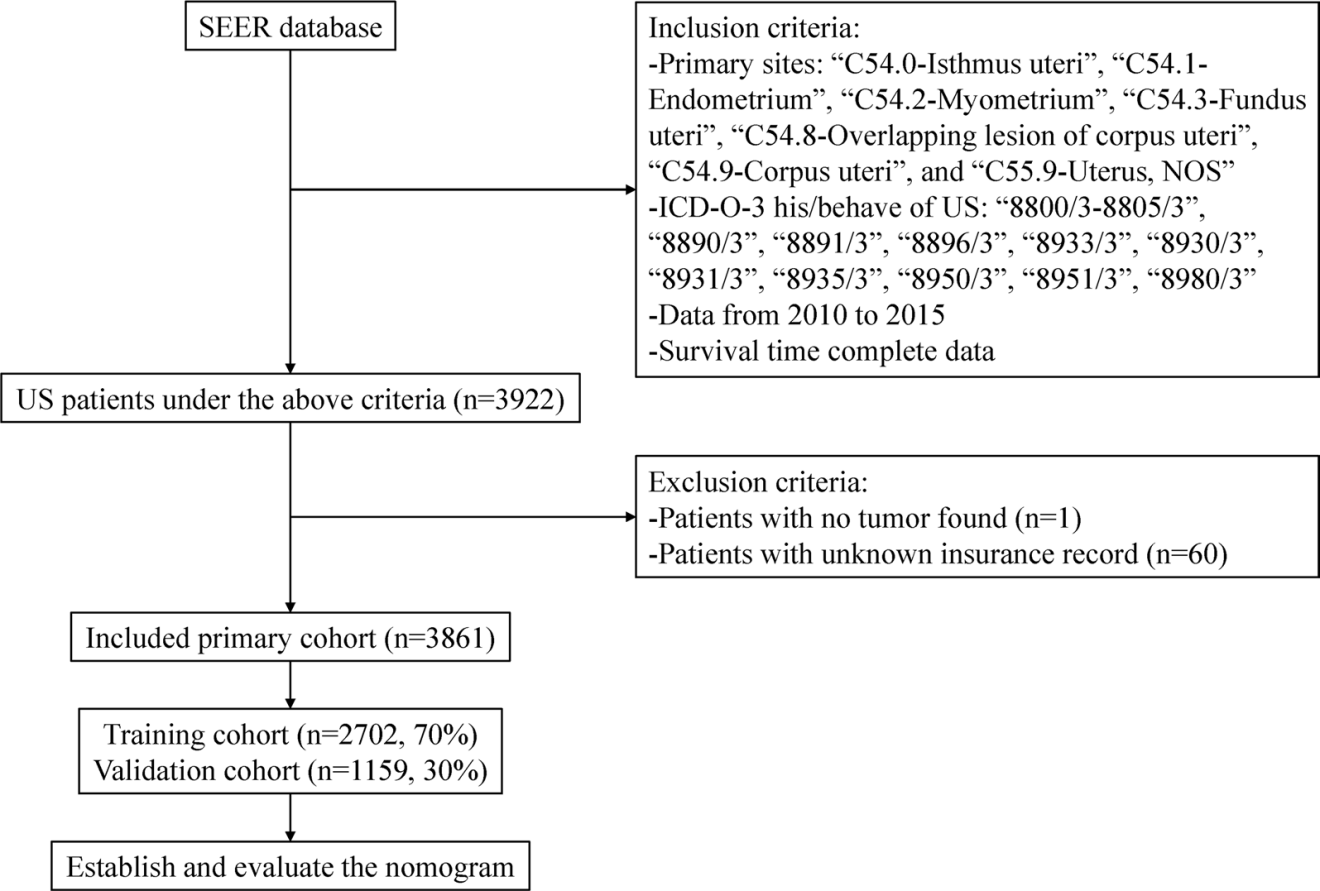
Patient selection flowchart. SEER, Surveillance, Epidemiology, and End Result Program; ICD-O-3, International Classification of Disease for Oncology, Third Edition

The exclusion criteria were follows: (1) patients with no confirmation by microscopy or only on autopsy; (2) no tumor found; and (3) unknown insurance status. This retrospective study initially identified 3922 uterine sarcoma patients enrolled in the SEER database from 2010 to 2015, among which 3861 patients were finally included based on the criteria mentioned above (Fig. 1).

### Statistical analysis

Twelve pathological and clinical features that we mentioned above were applied to conduct the analyses. All of the selected patients were randomly divided into a training cohort (n = 2702) and a validation cohort (n = 1159) at a ratio of 7:3 for nomogram construction. Continuous variables are presented as medians with interquartile ranges, while categorical variables are presented as numbers with percentages. The distributions of the characteristics between the training and validation cohort sets were compared using the χ^2^ test. Variables with statistical significance in the univariate analysis were entered into multivariate regression analysis. Cox regression was developed to assess the effects of correlation factors (*p* = 0.1). Then, we established a nomogram that predicted the 1-,3-, and 5-year CSS of US. The differentiation ability of the nomogram was evaluated using the C-index and the area under the time-dependent receiver operating characteristic (ROC) curve. A C-index value of 0.5 indicated no predictive power, and a C-index of 1.0 indicated complete differentiation. We compared the accuracy and comprehensiveness of our nomogram with the AJCC stage by using net reclassification improvement (NRI) and integrated discrimination improvement (IDI) [14]. The consistency of the survival probabilities predicted by the nomogram with the actual situation was assessed by calibration curves, and the clinical validity of the nomogram was tested via DCA [15].

All statistical analyses and graphics were performed with R software (version 3.6.0; http://www.Rproject.org) and SPSS software (version 24.0, SPSS, Chicago, IL, USA), with *P* < 0.05 considered to indicate statistical significance.

## Results

### Patient characteristics

A total of 3861 US patients extracted from the SEER database were randomly divided into a training cohort (n = 2702) and a validation cohort (n = 1159) at a ratio of 7:3. The median age at diagnosis in the training and validation cohorts was 62 years (interquartile range, 53–69 years) and 60 years (interquartile range, 51–69 years), respectively. The majority of the patients were white (69.2 and 71.2%), married (47.6 and 48.3%), and insured (95.4 and 96.3%). Most of the tumors were of pathological grade III or IV and larger than 50 mm in both cohorts. Nearly half of the patients were histologically diagnosed with carcinosarcoma. The distribution of different SEER stages was very similar between the groups, with a slightly higher rate in the localized group (37.4 and 42.5%). Nearly half of the patients were in AJCC stage I (41.0 and 45.1%) and less than one tenth were in AJCC stage II (9.7 and 8.1%). Most of the patients received surgery (90.8 and 92.4%), with a few receiving radiotherapy and over half receiving chemotherapy in both cohorts.

A summary of these clinical characteristics is given in Table 1.

**Table 1:** Patient characteristics in the study

Variable	Training cohort	Validation cohort
	(n = 2702)	(n = 1159)
Medium age at diagnosis, (25th–75th percentile)	62 (53–69)	60 (51–69)
Race n (%)		
White	1869 (69.2)	808 (71.2)
Black	591 (22.1)	243 (20.2)
Other	242 (8.6)	108 (8.6)
Marital status n (%)		
Married	1257 (47.6)	561 (48.3)
Single	586 (22.3)	261 (22.7)
SDW	736 (25.6)	293 (25.5)
Unknown	123 (4.6)	44 (3.5)
Insurance record n (%)		
Yes	2584 (95.4)	1117 (96.3)
No	118 (4.6)	42 (3.7)
Tumor size n (%)		
≤ 50 mm	710 (23.1)	313 (23.8)
> 50 mm	1596 (61.4)	677 (61.6)
Unknown	396 (15.6)	169 (14.6)
Pathological grade n (%)		
I	169 (6.4)	76 (6.0)
II	345 (12.0)	135 (11.4)
III	1193 (43.0)	527 (44.5)
IV	995 (38.7)	421 (38.2)
Histological type n (%)		
Sarcoma	76 (4.3)	20 (2.8)
Leiomyosarcoma	532 (28.3)	215 (26.6)
Adenosarcoma	124 (4.4)	45 (3.8)
Stromal sarcoma	455 (14.1)	207 (15.5)
Carcinosarcoma	1515 (48.8)	672 (51.3)
SEER stage n (%)		
Localized	1116 (37.4)	537 (42.5)
Regional	825 (29.1)	321 (28.2)
Distant	761 (33.5)	301 (29.3)
AJCC stage n (%)		
I	1225 (41.0)	573 (45.1)
II	236 (9.7)	84 (8.1)
III	572(20.0)	222 (20.0)
IV	669 (29.3)	280 (26.9)
Surgery status n (%)		
Yes	2516 (90.8)	1090 (92.4)
No/unknown	186 (9.2)	69 (7.6)
Radiotherapy status n (%)		
Yes	782 (26.3)	313 (24.6)
No/unknown	1920 (73.7)	846 (75.4)
Chemotherapy status n (%)		
Yes	1420 (52.4)	598 (53.2)
No/unknown	1282 (47.6)	561 (46.8)

### Variable screening and multivariate cox regression analysis results

According to the Cox stepwise regression analysis, tumor primary site, years of diagnosis and surgery site were excluded from further study due to no difference in the prognosis for US patients. Multivariate Cox regression analyses demonstrated that age at diagnosis [hazard ratio (HR) = 1.0116, *p* < 0.001], being black (HR = 1.1698, *p* < 0.05), single (HR = 1.2181 vs. married, *p* < 0.05), SDW (HR = 1.2965 vs. married, *p* < 0.001), tumor size (> 50 mm, HR = 1.4861 vs. ≤ 50 mm, *p* < 0.001; tumor size unknown, HR = 1.3345, *p* < 0.01), higher pathology grade(Grade III,HR = 7.3773 vs. pathology grade I, *p* < 0.001; IV, HR = 7.0185, *p* < 0.001), SEER stage (regional, HR = 1.8809 vs. localized, *p* < 0.001; distant, HR = 2.5199, *p* < 0.001), and higher AJCC stage (III,HR = 1.7459 vs. AJCC stage I, *p* < 0.001; IV,HR = 2.2275, *p* < 0.001) were all independent risk factors. Meanwhile, we found that leiomyosarcoma (HR = 0.6550 vs. sarcoma, *p* < 0.01), carcinosarcoma (HR = 0.6099 vs. sarcoma, *p* < 0.01), insurance (no insurance HR = 1.4851, *p* < 0.01), receiving surgery (no/unknown HR = 2.7559, *p* < 0.001), adjuvant radiotherapy (no/unknown HR = 1.3267, *p* < 0.001), and adjuvant chemotherapy (no/unknown HR = 1.5355, *p* < 0.001) were protective factors for surviving US. These results also indicated that other races, marital status, pathology grade II, adenosarcoma, stromal sarcoma and AJCC stage II were not significant risk factors (*P* > 0.05). The results of the multivariate Cox regression analysis are presented in Table 2.

**Table 2:** Selected variables in the SEER database by multivariate Cox regression analysis (training cohort)

Variable	Hazard ratio	95% CI	*p* value
Age at diagnosis	1.0116	1.0061–1.0172	< 0.001***
Race			
White	Reference		
Black	1.1698	1.0225–1.3383	0.022*
Other	0.8481	0.6698–1.0739	0.171
Marital status			
Married	Reference		
Single	1.2181	1.0436–1.4217	0.012*
SDW	1.2965	1.1230–1.4969	< 0.001***
Other	1.2816	0.9740–1.6863	0.076
Insurance record			
Yes	Reference		
No	1.4851	1.1367–1.9404	0.004**
Tumor size			
≤ 50 mm	Reference		
> 50 mm	1.4861	1.2674–1.7425	< 0.001***
Unknown	1.3345	1.0802–1.6487	0.007**
Pathological grade			
I	Reference		
II	1.3135	0.7662–2.2518	0.321
III	7.3773	4.5441–11.9771	< 0.001***
IV	7.0185	4.3528–11.3165	< 0.001***
Histological type			
Sarcoma	Reference		
Leiomyosarcoma	0.6550	0.4839–0.8866	0.006**
Adenosarcoma	0.6288	0.3919–1.0089	0.054
Stromal sarcoma	0.9274	0.6623–1.2984	0.661
Carcinosarcoma	0.6099	0.4535–0.8202	0.001**
SEER stage			
Localized	Reference		
Regional	1.8809	1.3831–2.5580	< 0.001***
Distant	2.5199	1.6708–3.8005	< 0.001***
AJCC stage			
I	Reference		
II	1.0136	0.7096–1.4478	0.941
III	1.7459	1.2843–2.3735	< 0.001***
IV	2.2275	1.4818–3.3484	< 0.001***
Surgery status			
Yes	Reference		
No/unknown	2.7559	2.2503–3.3751	< 0.001***
Radiotherapy status			
Yes	Reference		
No/unknown	1.3267	1.1567–1.5216	< 0.001***
Chemotherapy status			
Yes	Reference		
No/unknown	1.5355	1.3509–1.7453	< 0.001***

*SDW* Separated, divorced, and widowed, *SEER* Surveillance, Epidemiology, and End Results, *HR* hazard ratio, *AJCC* American Joint Committee on cancer

^*^p < 0.05, **p < 0.01, ***p < 0.001

### Nomogram construction

**Fig. 2:**
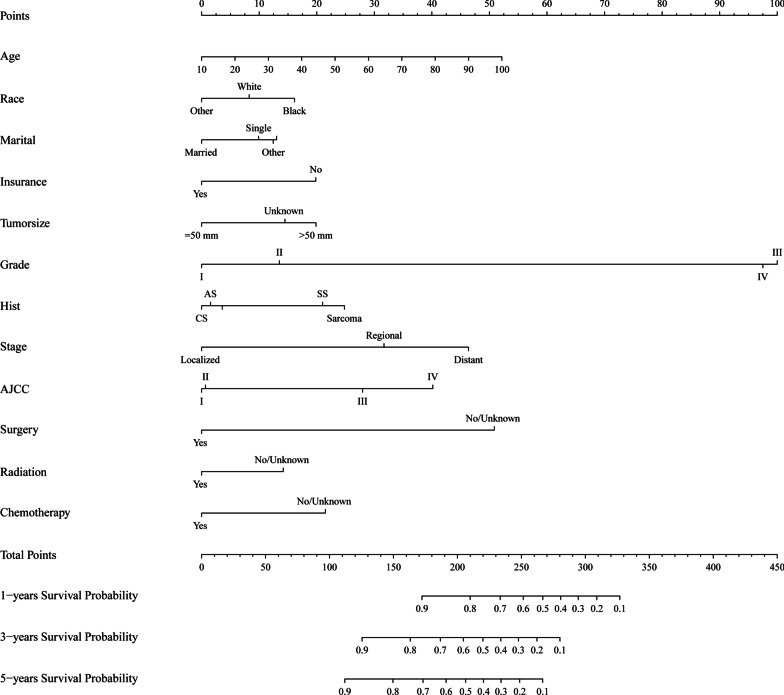
The nomogram for predicting 1-, 3- and 5-year survival of US. AJCC, 7th AJCC tumor stage

A nomogram was constructed for predicting the 1-, 3-, and 5-year CSS based on the multivariate Cox regression analysis (Fig. 2). The pathological grade was set as a reference scale ranging from 0 to 100 because it had the largest coefficient absolute value. The probabilities of 1-, 3- and 5-year CSS could be easily calculated by adding the point value of each variable. As shown in Fig. 2, the pathological grade had the strongest influence on CSS, followed by age at diagnosis, surgery status, SEER stage, AJCC stage, histological grade, chemotherapy, insurance record, tumor size, race, radiotherapy, and marital status.

### Nomogram comparison and evaluation

**Fig. 3:**
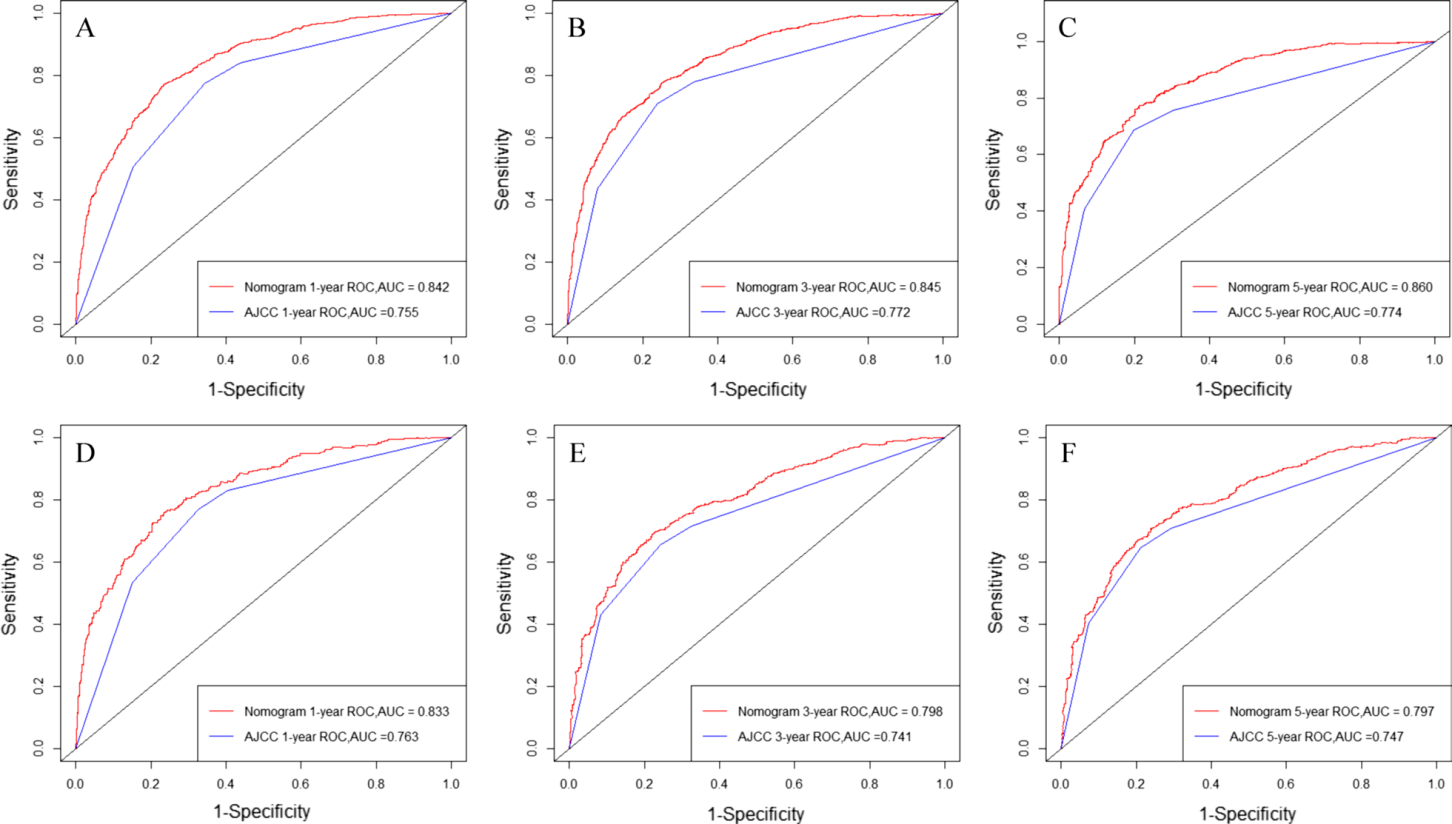
ROC curve analyses were generated to test the performance evaluation between the new model and the traditional AJCC model, by the AUC. **A**, **B** and **C** came from the training set, and **D**, **E**, and **F** came from the validation set

The C-indices in both the training and validation cohorts were higher than those of the AJCC 7th edition staging system (0.796 vs. 0.706, 0.767 vs. 0.713), indicating that our new model showed better discriminative ability. Furthermore, the AUCs of the training cohort for the 1-, 3-, and 5-year CSS were significantly larger (0.842, 0.845, and 0.860, respectively) than those of the traditional system (0.755, 0.772, and 0.774, respectively). Likewise, the ROC values were also significantly larger (0.833, 0.798, and 0.797 at 1-, 3-, and 5-year vs. 0.763, 0.741, and 0.747, respectively) (Fig. 3).

### Validation and calibration of the nomogram

The NRI values for the 1-, 3- and 5-year CSS rates in the training cohort were 64.6% (95% confidence interval [CI] = 55.3–73.5%), 59.0% (95% CI = 50.7–67.9%) and 62.2% (95% CI = 52.8–71.4%), respectively, and in the validation cohort, they were 47.2% (95% CI = 25.0–63.1%), 37.6% (95% CI = 14.1–51.4%) and 29.9% (95% CI = 7.4–55.0%), respectively. Moreover, the IDI values for the 1-, 3- and 5-year CSS rates in the training cohort were 8.64, 9.63 and 9.50%, respectively, and 3.98, 5.79 and 5.88% in the validation cohort (all *p* < 0.001). These results all indicated that the nomogram predicted survival with greater accuracy than the AJCC model.

**Fig. 4:**
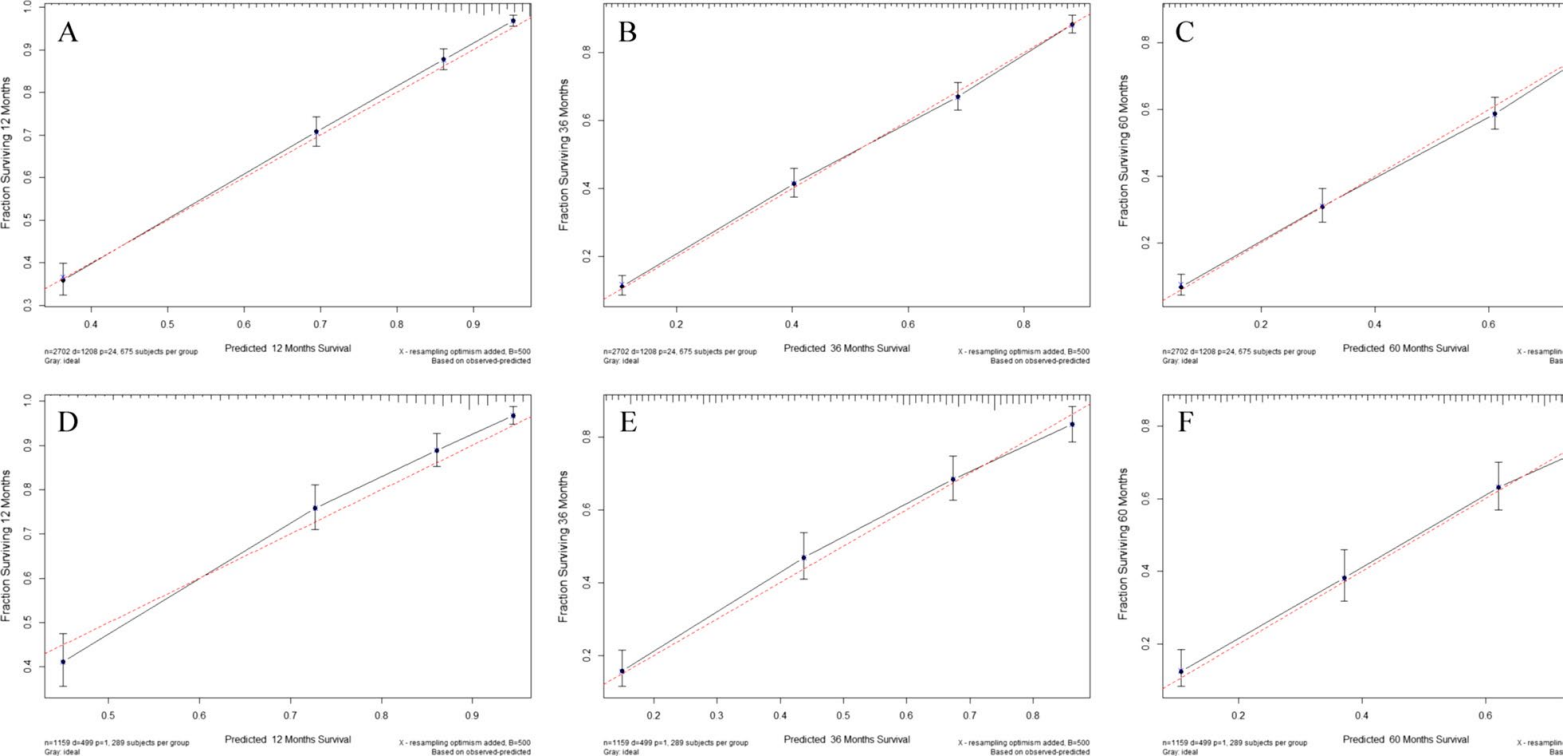
Calibration curves for 1-, 3- and 5-year CSS depict the calibration of each model in terms of the agreement between the predicted probabilities and observed outcomes of the training cohort (**A**, **C**, **E**) and validation cohort (**B**, **D**, **F**)

Calibration plots predicting the 1-, 3- and 5-year CSS for the training and validation cohorts were nearly identical to the practical observations, which demonstrated that the new model had excellent calibration ability (Fig. 4).

### Clinical usefulness

**Fig. 5:**
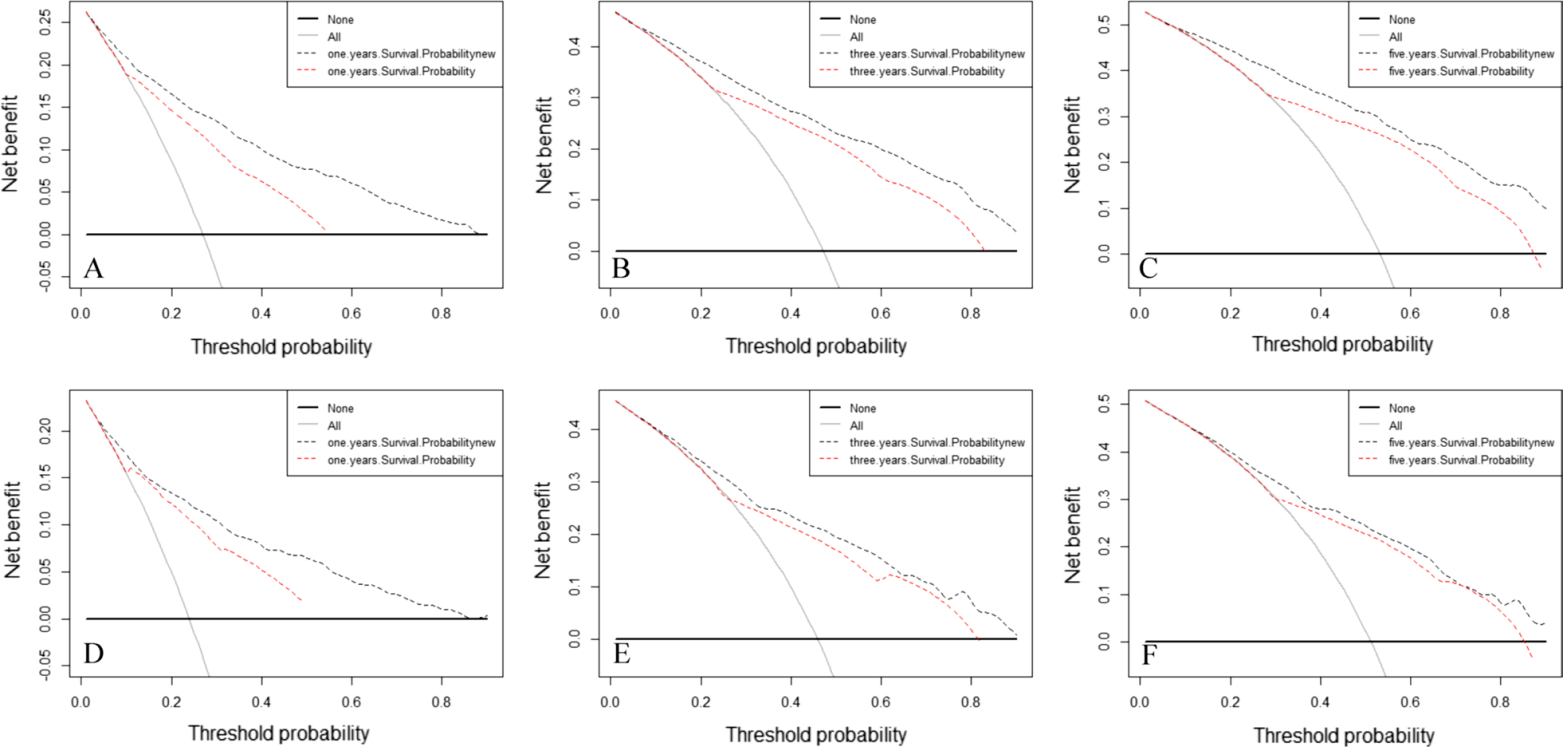
Decision curve analysis of the training (**A**, **B**, **C**) and validation cohorts (**D**, **E**, **F**)

Finally, a DCA curve was used to evaluate the clinical usability and benefits of the nomogram. Compared to the AJCC staging system, all of the DCA curves showed larger net benefits of the new model in both the training and validation cohorts (Fig. 5). These results demonstrated that the new model had better clinical benefits than the AJCC staging system.

## Discussion

Uterine sarcoma is a group of rare malignant mesenchymal tumors with various histologic types and aggressive progression and a poor prognosis [3]. To date, there is no efficient prognostic staging system that could help to estimate CSS at diagnosis for US patients.

A nomogram is a statistical tool that can provide accurate predictions for malignant tumors through a simple graphical presentation. A nomogram could provide accurate individualized predictions based on specified data points. Recently, nomograms have been developed for several cancers, such as NSCLC hepatocellular carcinoma (HCC), and adult skin melanoma [16–18]. However, few nomograms have been constructed for US patients. Zhou et al. [19] identified a 6-gene-based prognostic signature for US. Li et al. [20] evaluated the benefit of adjuvant radiotherapy for uterine leiomyosarcoma and carcinosarcoma. The latest and largest study performed by Mona Hosh et al. [10] identified 13,089 cases of uterine sarcoma diagnosed from 2000 to 2012. To our knowledge, this study was the first to develop a comprehensive prognostic nomogram to predict the 1-, 3-, and 5-year CSS for US based on the SEER database.

Pathological grade, age, surgery, AJCC stage, SEER stage, histological differentiation, chemotherapy, insurance record, tumor size, ethnicity, radiotherapy and marital status were identified as prognostic factors of CSS through multivariate Cox regression. Among them, the most notable factor affecting the CSS of US patients is pathological grade. The survival rates of patients with grades III and IV were worse than those of grade I patients, consistent with previous studies [21]. However, there were no significant differences between patients with grade III and grade IV disease.

Age at diagnosis had secondary crucial role in our model, although the exact mechanism remains unclear. Mona Hosh et al. [10] also found that the incidence of US increased with increasing age, and patients aged 50 years or older had worse survival than younger patients. The present study indicated that black US patients, tumor size (> 5.0 cm) and histological type were all associated with a poor prognosis for patients with US. Other studies also found that progression and poor survival rates were more common in patients who were black, had carcinosarcoma, or had larger tumors [22].

More interestingly, we discovered for the first time that marital status influenced the CSS of uterine sarcoma. Previous studies have shown that unmarried patients exhibit shorter OS and CSS than married patients withlung and liver cancer [23, 24]. In the present study, patients who were separated, divorced or widowed (SDW) had worse survival compared to married patients, followed by single patients. Marriage can not only provide financial support, but also relieve depression and anxiety among patients with cancer [25]. A spouse might influence the patients’ behavior, such as encouraging them to quit smoking and excessive alcohol drinking. Married patients are also more likely to accept more aggressive treatment and participate in regular follow-up. Thus, married patients would have better social support and better financial support, leading to a relatively better psychological state. Insurance was also strongly associated with the prognosis of uterine sarcoma. Patients with insurance could receive better medical support, be less economical and have less psychological distress than uninsured patients. This new information could help clinicians make more effective clinical decisions.

In addition, we also found that surgery status, SEER stage, AJCC stage and radiotherapy status affected the survival probability of US. Surgery is the gold standard treatment for US [2]. In addition, among these clinical parameters, the surgery status had the highest discriminating power in our study. Another important factor was the localized stage of US at initial diagnosis. Patients with metastatic disease at diagnosis had more aggressive disease than those with localized disease.

Radiation therapy is usually performed for advanced uterine sarcoma patients. Several retrospective studies suggested that radiotherapy after surgery could decrease pelvic recurrence, but not distant metastases [26]. Wong et al. [27] found that adjuvant pelvic radiotherapy might improve OS and reduce local recurrence for leiomyosarcoma. In our study, Fig. 2 clearly shows that both surgery and radiotherapy could improve survival base on the 1-, 3-, and 5-year CSS probabilities among US patients.

Notably, we identified for the first time that chemotherapy provides patients with a better prognosis. There are very few studies focusing on chemotherapy and patient prognosis for US patients in the SEER database. Efficacious chemotherapy to achieve prolonged survival in those with both early and advanced-stage US has been elusive. Hensley et al. [28] evaluated the role of 4 cycles of gemcitabine and docetaxel in 25 high-grade uterine leiomyosarcoma patients, and found prolonged PFS and OS. However, Littell et al. [29] compared gemcitabine-docetaxel with observation among 110 stage I uterine leiomyosarcoma (uLMS) patients after surgery, and found no significant difference in disease-free survival, OS or recurrence between the two groups. Our nomogram showed that chemotherapy had an even higher discriminatory power than radiotherapy. These data on chemotherapy could help clinicians choose individualized adjuvant treatment after surgery.

NRI, IDI, DCA, discrimination and calibration were used to evaluate the performance of the nomogram and to compare it with TNM-based AJCC staging. The survival nomogram showed better discrimination with C-indices of 0.796, and 0.767 for the training and validation cohorts, respectively, as the values were only 0.706 and 0.713 for the AJCC stage. As shown in Fig. 3, the 1-, 3-, and 5-year AUC values of the AJCC staging system were significantly lower than those of the nomogram. The plots resembling 45-degree lines indicated that the predictions of our nomogram were well-calibrated. Furthermore, the NRI and IDI both demonstrated that the new nomogram improved the predictive ability compared with the AJCC staging system. Finally, DCA curves were used to assess the clinical effectiveness of the nomogram. Our results showed that the 1-, 3-, and 5-year DCA curves for CSS exhibited better clinical effectiveness for predicting survival than the traditional AJCC staging system in both the training and validation cohorts.

This study, of course, still had several limitations. First, adjuvant hormonal therapy was not included in this nomogram, which might be because hormonal therapy is not routinely recommended as postoperative treatment in all histological types of US. Second, the SEER database did not use the FIGO staging system for US patients; instead, the SEER stage and AJCC stage were used. Third, some potential predictive variables, such as serum markers and neutrophil-to-lymphocyte ratios, were not included in this study because of the absence of these data absence in the SEER database. Fourth, our study excluded patients diagnosed after 2015. The NCCN guideline for uterine neoplasms has modified the pathology types of uterine sarcoma since 2016. More recently, diagnosed patients and patients with several rare pathological types were excluded to ensure sure sufficient follow-up so that we could adequately assess the association of treatment with survival.

## Conclusions

In summary, we have established a novel nomogram to predict the 1-, 3-, and 5-year CSS for US based on the SEER database. Our nomogram could be used as a valuable and effective tool to help clinicians to provide more individualized treatment and individualized survival prediction in clinical practice.

## Data Availability

Data from the SEER program is available for public. The data supporting the conclusions of this article are available in the Surveillance Epidemiology, and End Results (SEER) database (https://seer.cancer.gov/).

## Abbreviations

US: Uterine sarcoma
CSS: Cancer-specific survival
AJCC: American Joint Committee on Cancer
SEER: Surveillance, Epidemiology, and End Results database
SDW: Separated, Divorced and Widowed
C-index: Concordance index
AUC: Area under the curve
NRI: Net reclassification improvement
IDI: Integrated discrimination improvement
DCA: Decision-curve analysis
HR: Hazard ratio
ICD-O-3: International Classification of Diseases for Oncology, third edition morphology code
